# CD4 T Cell Metabolism Is a Major Contributor of HIV Infectivity and Reservoir Persistence

**DOI:** 10.20900/immunometab20200005

**Published:** 2020-01-10

**Authors:** Harry E. Taylor, Clovis S. Palmer

**Affiliations:** 1 Department of Microbiology & Immunology, SUNY Upstate Medical University, Syracuse, NY 13210, USA; 2 Life Sciences, Macfarlane Burnet Institute for Medical Research and Public Health, Melbourne, VIC 3004, Australia; 3 Department of Infectious Diseases, Monash University, Melbourne, VIC 3004, Australia

**Keywords:** HIV, metabolism, immunometabolism, HIV cure, immune activation, CD4 T cells

## Abstract

HIV infection is characterized by elevated glycolytic metabolism in CD4 T cells. In their recent study, Valle-Casuso et al. demonstrated that both increased glucose utilization and glutamine metabolism are essential for HIV infectivity and replication in CD4 T cells. Here, we discuss the broader implications of immunometabolism in studies of HIV persistence and their potential to inform new treatment and curative strategies.

## INTRODUCTION

Delivering antiretroviral therapy (ART) to treat HIV infection on a global scale is challenging. Thus, there is intense interest in developing strategies capable of achieving HIV eradication (“sterilizing cure”) or enabling indefinite ART cessation without virologic rebound (“functional cure”). Long-lived memory CD4 T cells harbouring transcriptionally active HIV (active reservoir) or integrated HIV DNA (latent reservoir) persist in patients on ART with full virologic control (HIV+ART+) and constitutes the major HIV reservoir. In addition, long-lived HIV-infected tissue-resident macrophages residing in organs such as the brain (microglia), gastrointestinal tract, and adipose tissue, along with non-memory CD4 T cells likely contribute to the HIV reservoir. These along with continued virus production particularly in lymphoid tissue despite ART [1], represent the key sources of viral rebound following cessation of ART. An increasing body of work has brought the role of immunometabolism in HIV pathogenesis into focus, together highlighting an intricate link between glucose metabolic disorder, activation status, and HIV permissivity in CD4 T cells [2–4]. These studies have expanded our understanding of immune activation and HIV replication and persistence considerably [5–8]. Nonetheless, the potential contributions of other nutrients in regulating of HIV replication remains incompletely understood.

## METABOLIC ACTIVATION OF CD4 T CELLS IS ESSENTIAL FOR HIV INFECTIVITY AND REPLICATION

Using a series of drugs that inhibit glycolysis, oxidative phosphorylation, fatty acid oxidation, and glutaminolysis, Valle-Casuso et al. confirmed the essential role of glucose metabolism in HIV reverse transcription and replication, but also discovered a potential reliance on glutamine uptake as well [9]. Interestingly, the hierarchy of susceptibility of CD4 T cells to HIV infection was associated with CD4 T cell metabolic activity, but independent of activation status. Effector memory CD4 T cells with the highest level of glucose uptake and basal metabolism were the most susceptible cells. In contrast, naïve T cells with the lowest metabolic activity were the least permissive to HIV infection. To determine whether the high metabolic activity of infected CD4 T cells was a cause or a consequence of HIV infection, the authors activated and sorted CD4 T cells based on their metabolic status prior to HIV challenge and successfully demonstrated a direct correlation between the metabolic activity and magnitude of infection of CD4 T cell subsets.

**Figure 1.**
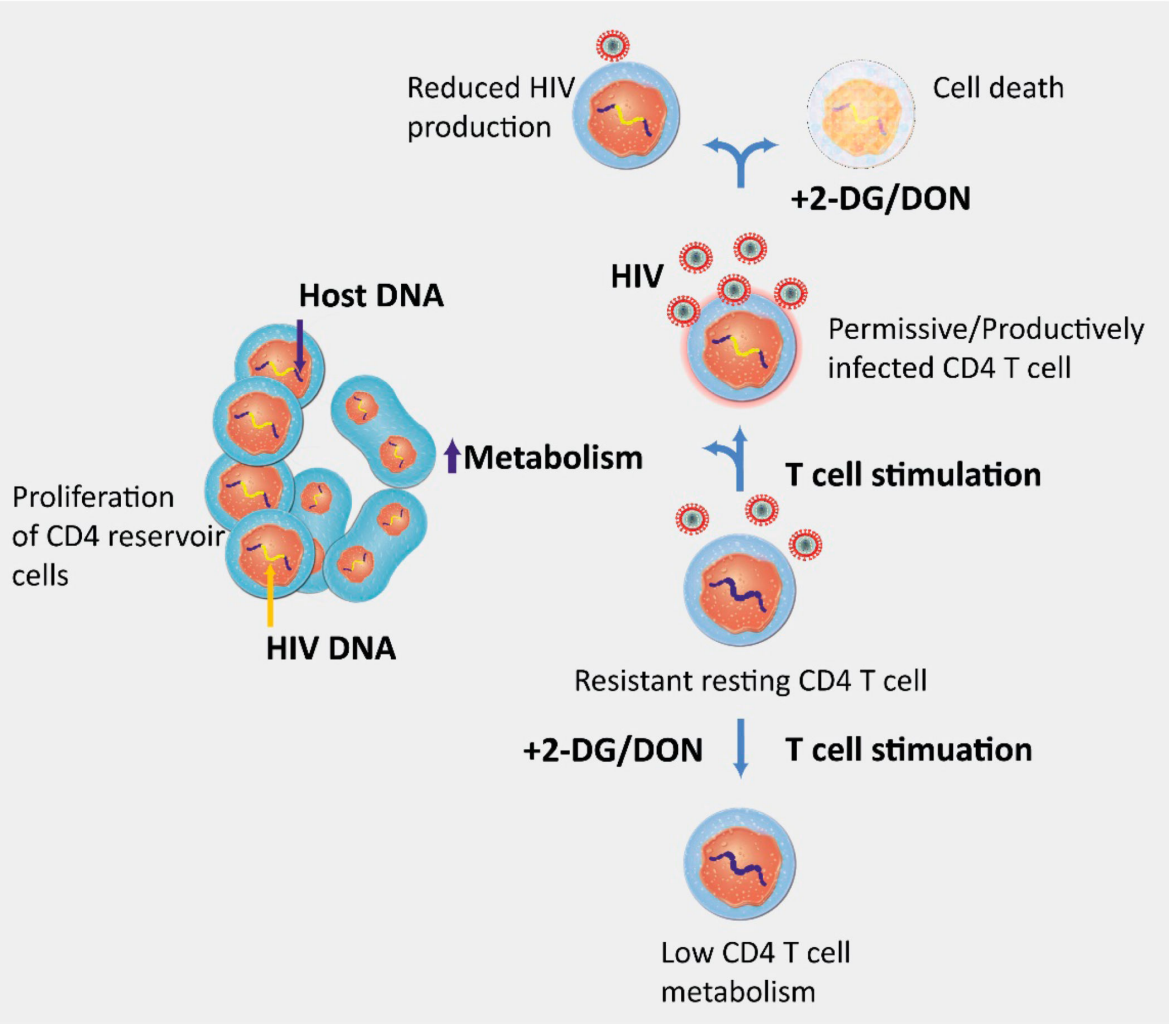
Valle-Casuso and colleagues showed susceptibility of CD4 T cells to HIV infection is dependent on CD4 T cell metabolic activity. Activation of CD4 T cells in the presence of drugs that block glycolysis (2-DG: 2-deoxy-glucose) or glutamine metabolism (DON: 6-Diazo-5-oxo-L-norleucine), renders CD4 T cells resistant to HIV, while inducing death of infected cells. Elevated metabolism while providing substrates for HIV transcription also deliver building blocks for nucleotide synthesis essential for proliferation of CD4 reservoir cells.

Interestingly, they also found that treatment of CD4 T cells with 2deoxy-glucose (2-DG), an inhibitor of glycolysis, impaired accumulation of HIV cDNA after HIV infection, suggesting inhibition of reverse transcription (RT) and a dependency of early replication on a highly glycolytic environment in CD4 T cells. Moreover, because the number of latently infected cells was reduced following inhibition of glucose metabolism, the authors concluded that a glycolytic phenotype was necessary for completing the pre-integration steps of HIV replication required for the establishment of latency in CD4 T cells. Furthermore, 2DG induced higher levels of cell death in HIV-infected CD4 T cells, when compared to uninfected cells. This is presumably a result of the elevated glycolytic dependence and activity of these cells, a feature of many “glucose-addicted” cancer cells. This Warburg phenotype also renders them vulnerable to killing by glycolytic inhibitors. Interestingly, CD4 T cells treated with 2-DG during activation are resistant to HIV infection (Figure 1).

## METABOLIC RESTRICTION OF HIV REPLICATION IN CD4 T CELLS

Resting CD4 T cells are highly resistant to productive HIV infection. Post-entry, reverse transcription of the viral RNA genome and subsequent nuclear transport of the resulting cDNA product are severely abrogated in these metabolically quiescent cells [10]. However, TCR engagement by anti-CD3/anti-CD28 stimulation was found to overcome this block and increase HIV infection of all CD4 T cell effector subsets to levels that directly correlated with their metabolic activity, not activation status at the time of infection. This finding is quite surprising, since HIV infection is known to depend on the activation state of CD4 T cells. It should be noted that in this study highly activated and low activated CD4 T cells were defined as CD25^+^/HLA-DR^+^ and CD25^−^/HLA-DR^−^, respectively. This is a major caveat, since these markers fail to accurately identify active and quiescent CD4 T cell populations. In fact, recent reports utilizing comprehensive mass cytometry analysis of cell cycle and activation marker expression in CD4 T cells show similar numbers of cycling (Ki-67^+^) cells in both populations [11]. This should serve as a call to the field to standardize the classification of resting versus activation phenotypes more rigorously. Indeed, memory CD4 T cells, conventionally defined as “resting” were found to express high levels of Glut1 in HIV+/ART+ individuals [6]. Glut1 is essential for CD4 T cell activation and HIV infection [5,12]. Nonetheless, the authors were able to unmask a link between HIV infection and T cell metabolism by using sub-optimal conditions of stimulation. Their observations added new mechanistic insights into the intricate relationship between metabolic status of CD4 T cells and susceptibility to HIV infection.

It is well known that activated T cells undergo global “metabolic reprogramming” to meet the biosynthetic demands of processes necessary for T cell effector functions [13]. For example, dNTP pools expand significantly during T cell activation to fuel DNA synthesis crucial for T cell clonal expansion. On the flip side, this increase in dNTP availability provides key building blocks that fuels HIV reverse transcription. Previous studies have demonstrated that T cell activation signals are coupled to dNTP pool expansion via a c-Myc-dependent transcriptional program which includes essential genes for glucose transport (Glut1/SLC2A1), glutamine transport (ASCT2/SLC1A5) and dNTP synthesis (RRM2) [14]. Remarkably, the authors found expression levels of Glut1, ASCT2, and RRM2 to positively correlate with HIV infection levels in CD4 T cell subsets. Glut1 and ASCT2 provide critical carbon (glucose) and nitrogen (glutamine) sources, respectively, for metabolic processes required for de novo nucleotide biosynthesis. Moreover, RRM2 is a critical subunit that forms the catalytic site of ribonucleotide reductase (RNR), the rate-limiting enzyme for dNTP biosynthesis. Inhibiting either glucose or glutamine metabolism were found to inhibit HIV infection, but blocking glucose metabolism was also found to inhibit RT, an observation consistent with suspected dNTP pool depletion in cells. It would be of interest to determine if dNTP pools were depleted with these treatments and if HIV replication could be rescued with exogenous nucleosides. Another finding that cellular metabolism serves as a rheostat that controls the susceptibility to HIV infection via metabolite pools, such as dNTPs, is highlighted by the fact that expression of SAMHD1, a HIV host restriction factor that depletes dNTP pools [10], in CD4 T cell subsets is negatively associated with infection. This suggests that naïve T cells, that exhibit low levels of glucose metabolism capable of fueling dNTP pool synthesis, also have the highest level of dNTP-depleting capacity, thereby potentially restricting HIV replication by two converging pathways. Surprisingly, SAMHD1 was the only restriction factor to demonstrate this association, although several factors have been identified that inhibit HIV replication in CD4 T cells. The activity level of some restriction factors is also regulated posttranscriptional, thus potentially explaining why more of these factors were not identified in gene expression analysis by Valle-Casuso et al.

This study raises new questions about how T cell metabolism governs susceptibility to HIV infection and pathogenesis. It also supports the recent findings by an independent group whereby glutamine by providing substrates for oxidative phosphorylation and nucleotide synthesis supported early steps of HIV infection in CD4 T cells [15]. Further work interrogating different targets critical for glutamine uptake and metabolism will shed more light on its role in HIV replication. We can be assured that these reports will provide fodder for new investigations aimed at dissecting the role of metabolism in HIV latency and persistence, as well as general studies of metabolism at the host-pathogen interface.
